# Stimulus Heterogeneity in a Task-Irrelevant Dimension Affects Selective Attention

**DOI:** 10.3390/bs13060495

**Published:** 2023-06-12

**Authors:** Cheol Hwan Kim, Suk Won Han

**Affiliations:** Department of Psychology, Chungnam National University, Daejeon 34134, Republic of Korea; cheol.hwan.kim2391@gmail.com

**Keywords:** stimulus-driven competition, task-irrelevant dimension, stimulus heterogeneity

## Abstract

When multiple stimuli are simultaneously presented, they compete against each other to be represented in the capacity-limited visual system. This competition increases as stimulus heterogeneity increases. Given that selective attention is a way to resolve this competition, it has been known that the effect of attention on task performance is magnified as the level of competition increases due to increased stimulus heterogeneity. While previous studies showed that stimulus heterogeneity in a task-irrelevant dimension affects task performance, it remains unknown how this kind of stimulus heterogeneity interacts with visual attention and stimulus-driven competition. Here, we found that the process of searching for a target stimulus among non-targets became inefficient as stimulus heterogeneity in a task-irrelevant dimension increased. The results also showed that the magnitude of the attentional cuing effect could be affected by increased heterogeneity. However, this modulation was dependent on the type of varied feature or task demand. We suggest that increased stimulus heterogeneity in a task-irrelevant dimension would increase stimulus-driven competition, which impoverishes the quality of stimulus representations.

## 1. Introduction

When multiple stimuli are presented in the visual field, selective attention enables a subset of them to be prioritized or processed in greater detail at the expense of others. According to the biased competition theory, multiple stimuli compete to be represented in the visual system, and suppress each other [1]. This stimulus-driven competition can be resolved when one of the presented stimuli receives attention in a bottom-up or top-down manner [1,2,3]. 

Notably, the mutual suppression between stimuli is well observed when the stimuli are heterogenous; a set of multiple homogenous stimuli do not evoke competitive interaction [1,2,4,5]. In line with this, several previous studies showed that when multiple heterogenous stimuli were presented, the process of searching for a target stimulus among other stimuli was severely compromised compared to when homogenous stimuli were presented [6,7,8,9]. 

Based upon these findings, Duncan and Humphrey (1989) formulated a theory claiming that the similarity between the target and non-targets, as well as the similarity between non-targets, affects visual search efficiency. In their model, visual search initiates with a parallel process of all search stimuli, followed by a selection process by matching input stimuli against an internal, target template. By this top-down control, stimuli similar to the target or items with target features are biased for selection, while stimuli dissimilar to the target would be suppressed. Similarly, the guided search model proposed by Wolfe and colleagues also claimed that following the initial parallel stage, top-down control separates potential target stimuli from less favorable stimuli [10,11]. Following this, a limited number of inputs are entered into the capacity-limited visual short-term memory, and at this stage, the target is identified. In this framework, high similarity between the target and non-target severely hampers search efficiency because the target and non-targets, similar to each other, are likely to compete for access to visual short-term memory. Another notable feature of this theory is that the similarity between the non-targets was also considered. Non-targets dissimilar to the target would be suppressed and this suppression would be easily spread when the non-targets are homogenous. Hence, the homogeneity/heterogeneity between non-target stimuli is also a significant factor for visual search efficiency.

What we aim to address in the present study is whether stimulus heterogeneity in a task-irrelevant dimension also affects visual search efficiency. In many previous search experiments, such as the experiments by Duncan and Humphreys, the stimulus heterogeneity was manipulated in the task-relevant dimension; the target was a particular shape while the non-targets were a homogenous or heterogenous set of shapes. In experiments reported here, we had participants search for an orientation-defined target (Experiment 1 and 2) or a shape-defined target (Experiment 3). Then, we varied the spatial frequency (Experiment 1) or the color of the stimuli (Experiment 2 and 3). Hence, the task-relevant dimensions were the orientation or shape, while the task-irrelevant features were the spatial frequency and color. Notably, we varied stimulus heterogeneity in a completely orthogonal dimension to the target-defining feature dimension. 

In this experimental setting, there could be two predictions regarding the effect of stimulus heterogeneity on visual search efficiency. First, visual search efficiency might be similar across the homogenous and heterogenous stimulus conditions. With the targets defined with a particular orientation or shape, an orientation-based or a shape-based target template would be formulated [7]. With this target template, any irrelevant feature dimension, such as color or spatial frequency, would be filtered out and not be considered for attentional selection. In a similar vein, separate feature maps would be created for each different feature dimension and selective attention would be operated only on the relevant feature map [11].

Alternatively, stimulus heterogeneity in a task-irrelevant dimension would affect visual search efficiency. Specifically, even though the task-irrelevant features need not to be attended, heterogenous stimuli would compete to be represented in the visual system, while such competition would be attenuated with a set of homogenous stimuli [2,5]. This competition would lead to mutual suppression between the stimuli, which can hamper the processing of each individual stimulus. 

While neither the feature similarity model nor the guided search model makes an explicit prediction regarding the effect of stimulus heterogeneity in a task-irrelevant dimension, recent studies suggested the possibility that such heterogeneity is an important factor that affects visual search performance [12,13]. In the study by Wei and colleagues, participants were instructed to search for orientation- or shape-defined targets. When the search stimuli were colored, given that the target was defined by orientation or shape, the color of the stimuli was a task-irrelevant feature. Their results showed that response times (RT) were shorter when all the search stimuli had the same color (homogenous) than when colors of the stimuli were heterogenous [13]. Similarly, Becker and colleagues also showed that increased stimulus heterogeneity in a task-irrelevant dimension impaired task performance [12]. In their experiment, the target was an outlined square with a gap either on the top or the bottom of the square, while the distractors had a gap either on the left or right side. Importantly, in one type of trial, search stimuli had two different colors, while in the other, five colors were used for the search stimuli. The results showed that increased heterogeneity in the task-irrelevant feature dimension (color) slowed search responses.

What is remarkable in these studies is that the researchers manipulated stimulus heterogeneity in a feature dimension that is completely orthogonal to the task-relevant dimensions; stimulus color varied under a shape or orientation search task. However, these studies did not directly investigate how the task-irrelevant stimulus heterogeneity interacts with visual attention. 

Indeed, there are some studies which investigated how stimulus heterogeneity of task-irrelevant distractors affects selective attention. In one such study [14], participants were instructed to search for a red tilted bar among white tilted bars and vertical red bars, while display items also differed in terms of size. In this case, the task-relevant features were the orientation and color of stimuli, while the item size was a task-irrelevant feature. Results demonstrated that when the size was correlated with other task-relevant features, search efficiency benefited. While this finding suggests that task-irrelevant features are also encoded into the visual system, this study did not directly manipulate stimulus homogeneity/heterogeneity. 

Expanding the previous studies, we investigated how stimulus heterogeneity in a task-irrelevant dimension interacts with visual attention. In the present study, we suggest that increased stimulus heterogeneity in a task-irrelevant dimension would potentiate mutual suppression between the stimuli. In this case, with heterogenous stimuli, the process of searching for a specific target stimulus would become less efficient than with homogenous stimuli. That is, the visual search slope, calculated by dividing the increase in search RT by the increase in search set size, would be steeper. 

While measuring the effect of task-irrelevant heterogeneity on visual search efficiency, we also examined whether such heterogeneity affects the effect of an attentional cue. Specifically, prior to the target search display, we presented a transient, salient stimulus. This kind of stimulus is well known to capture attention and induces enhanced processing of the cued location. Previous studies showed that the effect of the attentional cue was stronger when there were multiple items in the display [15,16,17,18]. This finding was interpreted to indicate that simultaneously presented stimuli evoke mutual competition and the attentional cue biases processing resources to the attended stimulus to resolve this competition [18]. Hence, we expected that the effect of an attentional cue would increase as the search set size increases. Furthermore, as we hypothesize that heterogenous stimuli in a task-irrelevant dimension evokes stronger competition than a set of homogenous stimuli, we examined whether the effect of the attentional cue would be greater for the heterogenous trials than for the homogenous trials in a task-irrelevant dimension. Notably, the previous studies reviewed above did not directly investigate how stimulus heterogeneity in a task-irrelevant dimension affects visual search efficiency or the attentional cuing effect.

Taken together, we investigated how stimulus heterogeneity in a task-irrelevant dimension interacts with visual attention. We examined this issue using two measures of visual attention: visual search efficiency and the attentional cuing effect. We hypothesized that a set of heterogenous stimuli in a task-irrelevant dimension would evoke greater stimulus-driven competition and mutual suppression than homogenous stimuli. This mutual suppression would impair the processing of each individual search item, lowering visual search efficiency. Furthermore, we also expected that increased mutual suppression would magnify the effect of an attentional cue, given the role of attention in resolving stimulus-driven competition. 

## Experiment 1

## Methods

### Participants

Sixteen adults (8 males, 18–25 years) with normal or corrected-to-normal vision participated for course credit. The Chungnam National University Institutional Review Board approved the experimental protocol and informed consent was obtained from each participant. 

Given that there were no previous studies addressing the effect of stimulus heterogeneity in a task-irrelevant dimension on search efficiency and the cuing effect, we considered previous studies yielding highly significant set-size effects and cuing effects. Specifically, we considered two datasets of our published studies. In such a study [18], N of 12 was sufficient to yield a significant cuing effect and set-size effect. In another study, N of 16 was used [19]. Using datasets of these studies, we calculated the effect size of cuing and set-size manipulations and ran a power analysis. The power analysis revealed that N of 16 was sufficient to yield a significant cuing effect and set-size effect at the power level of 0.95. Hence, we decided to collect data from 16 or more people.

### Stimuli and Apparatus

The experiment was programmed and run using PsychoPy3 [20]. The task stimuli were presented with a grey background. At the beginning of each trial, a black fixation dot was presented at the center of the screen throughout the experiment. Eight white outline squares (1.7 × 1.7° of visual angle with 0.03° of line thickness) were continuously present with the fixation to mark the locations where targets and distractors would be placed. The cue stimulus was a green outline square of the same size and line thickness as the place holders. The target was either a left- or right-tilted Gabor grating, whereas distractors were Gabor gratings with vertical orientation. These place holders were presented at the eight locations of an imaginary circle with a radius of 5.0 degrees. The contrast of the gratings was set to 100% and the spatial frequency of the gratings was 0.5, 1.0, 1.5, 2.0, 2.5, 3.0, 3.5, or 4.0 cycles per degree (c/deg).

### Design and Procedure

**Figure 1:**
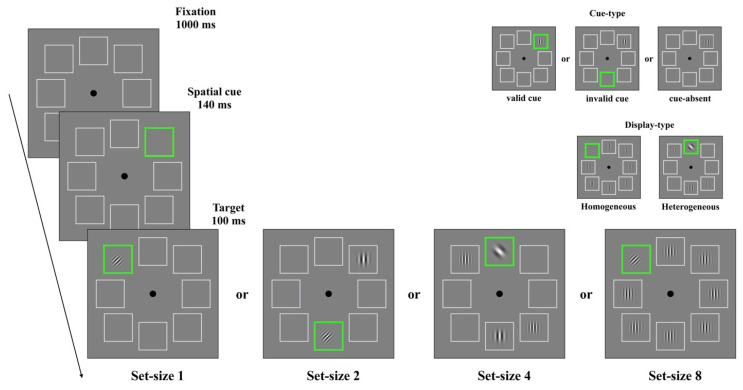
Trial design of Experiment 1.

As shown in Figure 1, the trial started with a 1000 ms fixation presentation, followed by the presentation of a cue that remained visible until the offset of the stimuli. There were three different types of cues: valid, invalid, and no cue. In the valid cue trials, a green outline square appeared at the place holder location that would contain the target. In the invalid cue trials, the cued location and target location did not match. In the cue-absent trials, the cue was not presented. The proportions of valid, invalid, and cue-absent trials were equal (1/3 for each). The target was tilted by 45°. The target stimulus was presented by itself or was accompanied by one, three, or seven distractors. In the homogeneous trials, the spatial frequencies of all the stimuli were the same, whereas in the heterogeneous trials, each stimulus had different spatial frequencies. The spatial frequency of a given stimulus was randomly chosen among 0.5, 1.0, 1.5, 2.0, 2.5, 3.0, 3.5, or 4.0 cycles per degree. The target and distractors were presented after the onset of the cue and remained visible for 100 ms. The cue-target SOA was 140 ms. For cue-absent trials, in which no cue was presented, the task display appeared 1140 ms after the fixation presentation. Participants were instructed to indicate the orientation of the grating via pressing the F key (left) or the J key (right) on the keyboard. Immediately after responses, the fixation was presented. Hence, the inter-trial interval was 1000 ms.

Taken together, the experiment consisted of a 3 × 2 × 4 factorial design, with cue type (valid, invalid, and cue-absent), task display (homogeneous and heterogeneous) and set size (set sizes of 1, 2, 4, and 8) as within-subject factors. Different types of cues, task displays, and set sizes were randomly intermixed within a single experimental block. There were twelve experimental blocks, each of which was made up of 192 trials.

## Results

**Figure 2:**
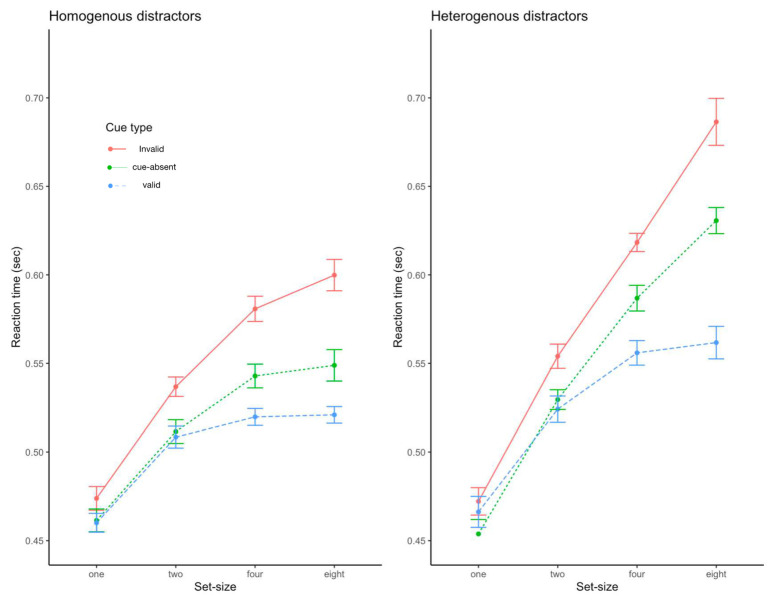
Reaction time data from Experiment 1. Error bars represent standard errors of the mean.

The results of Experiment 1 are shown in Figure 2. The overall proportion of correct responses was over 95%, which did not differ across trial types. To analyze the data, the RT data were entered into a repeated measures three-way ANOVA with cue type (valid, invalid, and cue-absent), task display (homogeneous and heterogeneous), and set size (set sizes of 1, 2, 4, and 8) as factors. Only trials with correct responses were included in the analysis. This analysis revealed a significant main effect of the cue type, F(2, 30) = 74.42, *p* < 0.001, η^2^ = 0.83, with the fastest responses for the valid and slowest responses for the invalid trials. We also found significant main effects of task display, F(1, 15) = 152.8, *p* < 0.001, η^2^ = 0.91, and set size, F(3, 45) = 112, *p* < 0.001, η^2^ = 0.88; RTs were greater for heterogenous trials and increased as set size increased.

The two-way interactions between cue type and set size, F(6, 90) = 22.35, *p* < 0.001, η^2^ = 0.60, and between task display and set size were significant, F(3, 45) = 39.36, *p* < 0.001, η^2^ = 0.72. These results indicate that the effects of the attentional cue and stimulus heterogeneity increased as the set size increased. Notably, the two-way interaction between cue type and task display was not significant, F(2, 30) = 1.72, *p* > 0.1, while the three-way interaction was significant, F(6, 90) = 3.65, *p* < 0.001, η^2^ = 0.20. While the absence of significant two-way interaction between cue type and task display implies that the magnitude of cuing was similar between the heterogenous and homogenous trials, the significant three-way interaction warranted further examination. Subsequent analyses revealed that the magnitude of the cuing effect was not significantly different between the homogenous and heterogenous trials when a modest number of stimuli (set sizes of 1, 2 and 4) were presented, *p*’s > 0.10. For a set size of 8, however, the cuing effect was significantly greater for the heterogeneous trials than for the homogeneous trials, t(15) = 3.75, *p* < 0.001.

## Discussion

A notable finding of the present experiment is that visual search efficiency was significantly lower for the heterogenous trials than for the homogenous trials, as revealed by the significant two-way interaction between set size and task display. This is consistent with our prediction that stimulus heterogeneity in a task-irrelevant dimension would impair visual processing of each individual search stimulus. Furthermore, we also found that with a relatively large set size, the cuing effect was greater for the heterogenous trials than for the homogenous trials.

## Experiment 2

In Experiment 1, we found that increased stimulus heterogeneity in a task-irrelevant dimension (spatial frequency) lowered visual search efficiency and increased the magnitude of the attentional cuing effect with relatively large set sizes. In this experiment, we examined whether such modulation would also be observed when the heterogeneity of a different feature dimension, color, was manipulated.

## Methods

### Participants

A separate group of eighteen adults (10 males, 18–25 years) with normal or corrected-to-normal vision participated in the study for monetary compensation. All experimental procedures were approved by the Chungnam National University Institutional Review Board. Informed consent was obtained from each participant.

### Stimuli and Apparatus

All stimuli and apparatus used were identical to those of Experiment 1 except for the following: all participants were tested online. The task stimuli were colored Gabor gratings presented with a black background. The color of the Gabor grating was randomly chosen from a selection of eight colors (red, blue, yellow, magenta, cyan, purple, orange, and brown), each of which was produced by RGB permutations. Eight grey outline squares (1.7 × 1.7 degree of visual angle with 0.1 degree of line thickness) were continuously present with a grey fixation to mark the locations where targets and distractors would be placed. The spatial frequency of the gratings was set to 2.0 cycles per degree (c/deg).

### Design and Procedure

**Figure 3:**
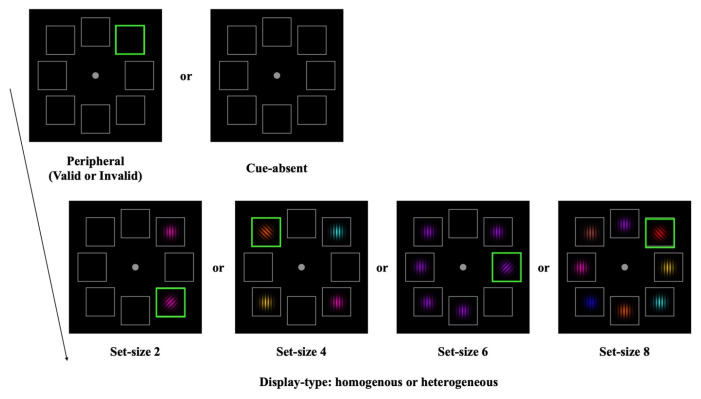
Trial design of Experiment 2. For the homogeneous trials, the color of each stimulus was the same, while all the stimuli had a different color for the heterogeneous trials.

The experimental design and procedure were identical to those of Experiment 1, with the following exception. As shown in Figure 3, the target stimulus was accompanied by one, three, five, or seven distractors. Hence, the levels of the set size were 2, 4, 6, or 8. We chose these set sizes because the cuing effect was modest for the set size of 1. Hence, we decided to replace this set size with a set size of 6, which was not included in Experiment 1. The color of each stimulus was randomly selected from a pool of eight colors (red, blue, yellow, magenta, cyan, purple, orange, and brown). In homogenous trials, the target and distractors were the same color, while in heterogenous trials, each stimulus had a unique color.

Notably, all participants were tested online. The participants were instructed to download and install the Psychopy software, and we sent the experimental script. To ensure that all the stimuli were presented in similar sizes and eccentricity for all participants, we instructed them to adjust their monitor resolution and screen width according to their monitor specification and viewing distance (57 cm), which was consistent with the setting of the testing room. A researcher informed the participants how to set up the experimental environments and how to conduct the experiment. The researchers confirmed their monitor settings through an online meeting tool before the experiment to make sure that the participants set it up properly.

## Results

**Figure 4:**
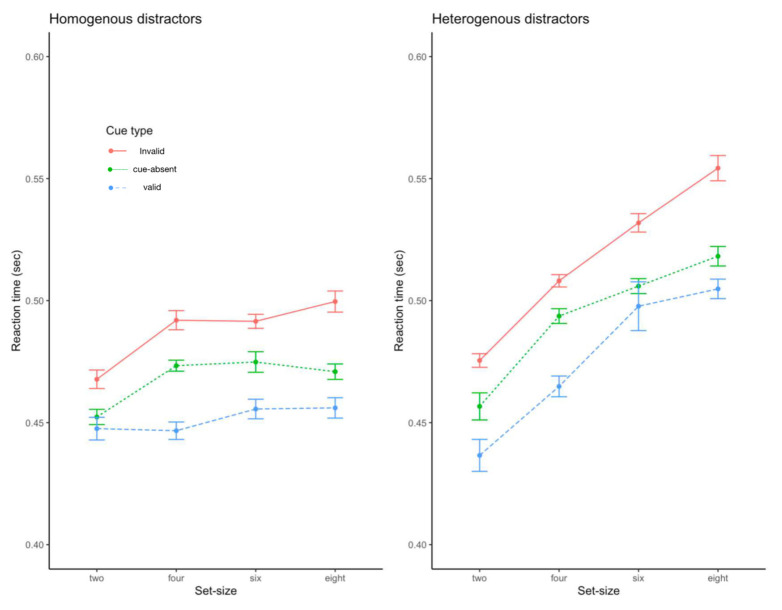
Reaction time data from Experiment 2. Error bars represent standard errors of the mean.

The analysis was performed in the same manner as Experiment 1 (see Figure 4). Response accuracy was over 92.5%, which did not differ across the trial types. The RT results showed that the main effect of the cue type was significant, F(2, 34) = 83.78, *p* < 0.001, η^2^ = 0.83, as were the main effects of task display, F(1, 17) = 104.5, *p* < 0.001, η^2^ = 0.86, and set size, F(3, 51) = 63.69, *p* < 0.001, η^2^ = 0.79. The two-way interactions between cue type and set size, F(6, 102) = 4.20, *p* < 0.001, η^2^ = 0.20, and between task display and set size were both significant, F(3, 51) = 40.87, *p* < 0.001, η^2^ = 0.71. The direction of significant main effects and interactions were all consistent with the results of Experiment 1: the valid cue yielded significantly faster responses than other cues and the cuing effect increased as the set size increased. Importantly, the search set-size effect was greater for the heterogenous trials than for the homogenous trials. However, the two-way interaction between cue type and task display was not significant, F(2, 34) = 1.54, *p* > 0.23. Finally, the three-way interaction was not significant, F(6, 102) = 1.34, *p* > 0.25.

## Discussion

Consistent with Experiment 1, search efficiency was found to be affected by stimulus heterogeneity in the task-irrelevant dimension. In both experiments, the task-relevant feature was orientation. Regardless of whether the task-irrelevant dimension feature was spatial frequency or color, the search process became less efficient when the search stimuli were heterogenous in the task-irrelevant dimensions. Regarding the cuing effect, we did not find any difference in the cuing effect across different task displays; stimulus heterogeneity in color did not affect the magnitude of the cuing effect. It is unclear about the reason for this discrepancy. Further research would be fruitful to unveil this issue.

## Experiment 3

In Experiment 3, we examined the effect of stimulus heterogeneity in a task-irrelevant dimension under a visual search task, employing real-world objects: cars. We had participants look for a specific type of car among non-target cars. The target could be identified by focusing on the shape. The colors of the car, a task-irrelevant feature, were heterogenous or homogenous.

## Methods

### Participants

Eighteen adults (6 males, 18–25 years) with normal or corrected-to-normal vision participated in the study for monetary compensation. These participants did not participate in any of the previous experiments in this study. All experimental procedures were approved by the Chungnam National University Institutional Review Board. Informed consent was obtained from each participant.

### Stimuli and Apparatus

All stimuli and apparatus used were identical to those of Experiment 2, except for the following: the target was a colored compact car or a sedan, while distractors were colored trucks. The color of the car was randomly chosen from a selection of eight colors (red, blue, yellow, black, silver, white, orange, and green). In this experiment, the background, fixation, and place holders were identical to those of Experiment 1.

### Design and Procedure

**Figure 5:**
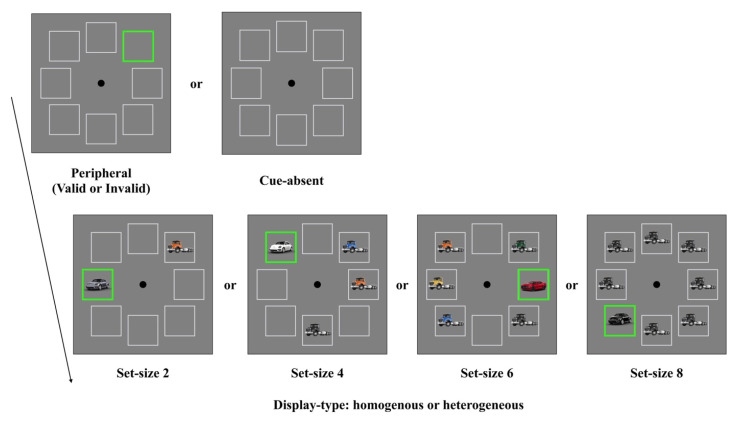
Trial design of Experiment 3.

The experimental design and procedure were identical to those of Experiment 2, with the following exceptions. As shown in Figure 5, the color of each stimulus was randomly selected from a pool of eight colors (red, blue, yellow, black, silver, white, orange, and green). Participants were required to identify whether the target was the compact car or the sedan. Given that complicated, real-world objects were used, we presented the search display until participants responded.

## Results

**Figure 6:**
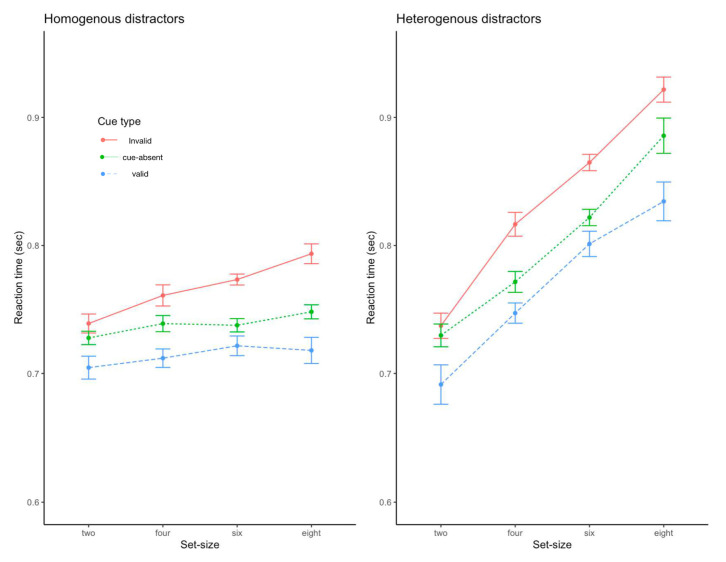
Reaction time data from Experiment 3. Error bars represent standard errors of the mean.

Response accuracy was over 94.5%, which did not differ across the trial types. As shown in Figure 6, the RT analysis revealed that the main effect of the cue type was significant, F(2, 34) = 35.44, *p* < 0.001, η^2^ = 0.68, as were the main effects of task display, F(1, 17) = 41, *p* < 0.001, η^2^ = 0.70, and set size, F(3, 51) = 79.15, *p* < 0.001, η^2^ = 0.82. The two-way interactions between cue type and task display, F(2, 34) = 3.43, *p* < 0.005, η^2^ = 0.17, between cue type and set size, F(6, 102) = 5.29, *p* < 0.001, η^2^ = 0.24, and between task display and set size were all significant, F(3, 51) = 34.17, *p* < 0.001, η^2^ = 0.67. The direction of those significant main effects and interactions was consistent with those of Experiment 1 and Experiment 2. Finally, the three-way interaction was not significant, F(6, 102) = 0.56, *p* > 0.76.

Importantly, we found that search efficiency was lower with heterogenous stimuli in the task-irrelevant dimension than with homogenous stimuli, consistent with Experiment 1 and Experiment 2. We also found that the cuing effect was significantly greater for the heterogenous trials than for the homogenous trials, as revealed by the significant two-way interaction between task display and cue type.

## Discussion

The results of Experiment 3 are clear. Under a visual search task using real-world objects, stimulus heterogeneity in a task-irrelevant dimension, color, affected visual search efficiency. We also found that the magnitude of the cuing effect was greater with heterogenous stimuli than with homogenous stimuli in the task-irrelevant dimension. This is a different pattern of results from those of Experiment 2. This discrepancy might be due to the fact that stimulus presentation was extended until participants responded in Experiment 3, whereas stimulus presentation duration was brief (100 ms) in Experiment 2. These results suggest the possibility that the encoding duration of task-irrelevant features is different for different types of features. Alternatively, the use of complicated, real-world objects might have amplified the effect of the color dimension on the attentional cuing effect. Related to this, the results of Experiment 1 also showed the significant impact of stimulus heterogeneity on the magnitude of the cuing effect. However, such modulation was dependent on search set size in Experiment 1; in Experiment 3, the effect of heterogeneity on the cuing effect was similar across set sizes. There are multiple factors to be considered to explain this discrepancy, such as the types of tasks and manipulated feature dimensions. Further research might be fruitful to clarify this issue.

## 2. General Discussion

The present study investigated how stimulus heterogeneity in a task-irrelevant dimension affects attentional processing. Importantly, we manipulated the heterogeneity of a feature dimension, the spatial frequency or color of visual stimuli, which was completely orthogonal to the task-relevant dimension: stimulus orientation or stimulus shape. We found that the process of searching for a target stimulus was inefficient when the stimuli were heterogenous in the task-irrelevant dimension Furthermore, under some circumstances, the magnitude of the attentional cuing effect was greater when heterogenous stimuli were presented than when a set of homogenous stimuli were presented. 

Specifically, in Experiment 1 and 2, participants performed the task of searching for a tilted Gabor grating among vertical gratings. A notable finding of Experiment 1 is that when a relatively large number of non-targets were present, the attentional cuing effect was larger for the heterogenous trials than for the homogenous trials. Furthermore, visual search efficiency was lower with heterogenous trials than with homogenous trials. However, in Experiment 2, while visual search efficiency was lowered when the stimuli were heterogenous in another task-irrelevant dimension (color), the magnitude of the cuing effect was not affected. Finally, we employed a visual search task using real-world objects in Experiment 3. The results showed that the cuing effect was significantly greater for the heterogeneous trials than for the homogeneous trials. Furthermore, consistent with the results of Experiment 1 and 2, search efficiency was lowered when the stimuli were heterogenous in the task-irrelevant dimension (color).

In all the experiments reported here, when search stimuli were heterogenous in a task-irrelevant dimension, visual search became less efficient than when the stimuli were homogenous. This was true regardless of the types of search stimuli (simple, controlled stimuli or real-world objects), manipulated types of irrelevant-features (spatial frequency or color), or stimulus presentation duration (100 ms or unlimited duration). Hence, we are confident in our claim that stimulus heterogeneity in a task-irrelevant dimension is a crucial factor for visual search efficiency. Indeed, a large number of previous studies also have shown that distractor heterogeneity affected visual task performance [6,7,12,13]. However, some of these studies did not specify how stimulus heterogeneity interacts with visual attention. In other studies, stimulus heterogeneity was manipulated in the task-relevant dimension. The important novelty of the present study lies in the fact that the stimulus heterogeneity was manipulated in a completely orthogonal dimension to the task-relevant dimension, and we examined how this factor affects visual search efficiency and the cuing effect. Notably, increased stimulus heterogeneity in the task-relevant dimension would have imposed no additional task demand, but this manipulation lowered visual search efficiency, and in some circumstances, the attentional cuing effect was magnified.

Contrary to the reliable effect of stimulus heterogeneity in a task-irrelevant dimension on search efficiency, the effect of stimulus heterogeneity on the attentional cuing effect differed, depending on the type of feature dimension manipulated and set size. When the spatial frequency of the search stimuli was heterogenous, a significantly greater cuing effect was observed with a large set size than when the stimuli had the same spatial frequency. Within the same task, the cuing effect was not affected by stimulus heterogeneity when a different feature dimension, color, was manipulated. However, when the search display duration was extended until response, with the task using complex, real-world objects, increased heterogeneity in the task-irrelevant dimension, color, increased the magnitude of the cuing effect across all set sizes. Certainly, this dissociation could be due to the different nature of visual processing for each different type of feature dimension. While this would be an important topic for further research, extensive discussion of this issue is beyond the scope of the present study.

To account for the effects of stimulus heterogeneity on visual search efficiency, we surmise that heterogenous stimuli in a task-irrelevant dimension compete against each other for limited processing resources to a greater extent than homogenous stimuli. This account is supported by neuroimaging studies showing that multiple heterogenous stimuli evoked greater suppressive interactions than homogenous stimuli [2,5]. Specifically, in the study by Shim and colleagues, while participants focused attention on the center of visual display, task-irrelevant images of faces, objects, or scenes were presented in the periphery. While participants did not have to pay attention to the face or scene images, those stimuli competed to be represented in the visual system; the simultaneous presentation of multiple stimuli evoked less activity in the visual cortex than the sequential presentation of the stimuli. Importantly, this mutual suppression was greater when heterogenous stimuli were presented than when homogenous stimuli were presented. With this increased suppression, processing of each individual stimulus would suffer. Furthermore, in some cases, to resolve this suppressive competition, a stronger bias toward a salient, attentional cue might be evoked, yielding an increased cuing effect.

This account can complement the dominant visual search models, such as the feature similarity model [7] and guided search model [11]. The feature similarity model emphasizes the role of top-down control in the selection of relevant features and the suppression of irrelevant features. Similarly, the guided search model argues that top-down control biases attention towards specific features or combinations of features that are more likely to distinguish the target from distractors. Even though the irrelevant feature dimension would not be considered in establishing a top-down task set, we demonstrated the significant impact of stimulus heterogeneity in a task-irrelevant dimension on search efficiency. Presumably, in the process of separating non-targets from the target and suppressing the non-targets, homogenous non-targets in the task-irrelevant dimension might be able to be suppressed more easily.

A limitation of the present study is that we could not clarify the exact relationship between stimulus heterogeneity and the magnitude of the attentional cuing effect. There might be several distinct factors that modulate the influence of stimulus heterogeneity on the attentional cuing effect. We believe that future studies regarding this issue would be fruitful.

To conclude, we provide clear evidence that increased stimulus heterogeneity in a completely task-irrelevant dimension affects visual search efficiency, and under some circumstances, the effect of a cue orienting spatial attention. We suggest that increased heterogeneity in a task-irrelevant dimension increases stimulus-driven competition. This increased competition should be primarily bottom-up, because the top-down task set was not affected by the heterogeneity manipulation. This increased mutual suppression interacts with visual attention.

## Data Availability

Data and code will be shared upon request.
